# Pilot study determining the feasibility of implementing the Disadvantaged Populations eGFR Epidemiology Study (DEGREE) protocol, point-of-care field measurements and a new module on risk factors for chronic kidney disease of unknown origin in Hispanic outdoor workers

**DOI:** 10.1186/s12882-021-02288-z

**Published:** 2021-03-12

**Authors:** Erika Figueroa-Solis, David Gimeno Ruiz de Porras, George L. Delclos

**Affiliations:** 1 Southwest Center for Occupational and Environmental Health, Department of Epidemiology, Human Genetics and Environmental Sciences, The University of Texas Health Science Center at Houston (UTHealth) School of Public Health, Houston, TX USA; 2 Southwest Center for Occupational and Environmental Health, Department of Epidemiology, Human Genetics and Environmental Sciences, The University of Texas Health Science Center at Houston (UTHealth) School of Public Health in San Antonio, San Antonio, TX USA; 3 Center for Research in Occupational Health, Universitat Pompeu Fabra, Barcelona, Spain; 4 CIBER Epidemiología y Salud Pública, Madrid, Spain

**Keywords:** CKDu, DEGREE, Hispanic, Workers

## Abstract

**Background:**

To field test the Disadvantaged Populations eGFR Epidemiology (DEGREE) protocol, outdoor point-of-care (POC) testing for serum creatinine, and a new risk factor module on chronic kidney disease of undetermined origin (CKDu) in U.S. outdoor Hispanic workers.

**Methods:**

Fifty workers were interviewed in Houston (TX). DEGREE and CKDu questionnaires were completed indoors. Anthropometrics and paired blood samples for POC and laboratory assay were completed outdoors over two periods (November–December 2017, April–May 2018).

**Results:**

Administration of DEGREE and CKDu questionnaires averaged 10 and 5 min, respectively, with all questions easily understood. We observed high correlations between POC and IDMS creatinine (r = 0.919) and BUN (r = 0.974). The POC device would disable testing when outdoor temperatures were above 85 °F or below 65 °F; this was adjustable.

**Conclusions:**

Implementation of DEGREE and the new CKDu module was straightforward and well understood. The POC device performed well in the field, with some adjustment in methods when temperature readings were out of range.

## Background

Chronic kidney disease (CKD) is a global health problem that can be caused by diabetes, hypertension, glomerulonephritis, congenital abnormalities, or obstruction of the urinary tract, among other known diseases [1]. The hallmark measurement for renal insufficiency is the glomerular filtration rate (GFR), which is usually estimated (eGFR) from the serum creatinine level, obtained in whole blood samples. Based on the eGFR, a CKD case definition has been established by the ‘Kidney Disease: Improving Global Outcomes CKD Work Group’, which categorizes CKD into five stages (G1-G5) and two substages (G3a and G3b) based on eGFR. Categories G3a-G5 correspond to decreased eGFR (i.e., eGFR < 60 ml/min/1.73m^2^) [2]. CKD cases identified in high-income countries are typically associated with lifestyle-related risk factors, such as type II diabetes and hypertension [3]. However, over the past 10–15 years, CKD cases have been described in low- and middle-income countries that do not fit this “usual” CKD pattern. These unusual cases predominantly affect male agricultural workers, often in their 30s and 40s, and are associated with high mortality [3–5]. Their prognosis is poor due to delays in diagnosis and limited availability of therapy (i.e., dialysis or renal transplantation).

This chronic impairment of kidney function not associated with known risk factors or a specific histological diagnosis has been termed “CKD of undetermined cause” (CKDu), “CKD of non-traditional cause” (CKDnt) or “Mesoamerican nephropathy” (MeN), given the initial description of CKDu in Central America [6]. Clusters of a similar CKD have also been reported in Sri Lanka, India, Saudi Arabia, Egypt, Senegal, and, more recently, in the United States (U.S.) [1, 7, 8]. In Central America, CKDu is hypothesized to be associated with occupational and environmental exposures affecting young men working in lowland agricultural settings, most notably sugarcane harvesters [9]. To date, at least for Central American CKDu, suspected causes include a combination of exposure to high heat and humidity, inadequate hydration, high physical demands and, possibly, concomitant use of nephrotoxic nonsteroidal anti-inflammatory agents. Although attention has focused primarily on the sugarcane industry, there are other at-risk occupations and industrial economic sectors characterized by similar exposures reported in Central America [10, 11].

Most of the literature has described local experiences in different parts of the world, but differences in case definition, study design, sampling approach, and/or methods with limit study comparisons. Additionally, there is lack of characterization of the distribution of *normal* eGFR in the affected populations, which is likely to vary regionally. Moreover, the degree of bias in GFR estimates is affected by ethnicity and body weight/muscle mass, with ethnicity-adjusted equations still being highly variable around the world [1, 12]. Designed to facilitate international comparisons of eGFR, the ‘Disadvantaged Populations Estimated Glomerular Filtration Rate (eGFR) Epidemiology Study’ (DEGREE) was launched to determine the worldwide distribution of both normal and reduced eGFR through the use of the same protocol. DEGREE was especially aim to researchers in low- and middle-income countries, encouraging them to follow a standardized protocol consisting of a minimum core of demographic data and standardized measurement of serum creatinine analyzed using isotope dilution mass spectrometry (IDMS) in a laboratory [1]. To date, DEGREE has not been tested in either Central America, where most cases of CKDu have been reported, or in similar collectives of workers in the U.S.

Although DEGREE uses central laboratory analysis of creatinine, there may be a need for testing in remote geographic areas in hot/humid outdoor environments where obtaining and transporting biological specimens may be excessively cumbersome or subject to specimen deterioration. Thus, having a reliable real-time measure of renal function could be useful. Point-of-care (POC) testing is defined as a medical diagnostic test done near the patient with near-immediate real time results. This blood sample can be taken from a fingerprick or venipuncture. Although the gold standard for serum creatinine analysis is IDMS in a central laboratory, this requires obtaining and transporting a whole blood sample, taking 30 min or longer to complete. POC has the advantage of immediate results that do not require transport of specimens and which can enable fast medical decision-making. POC devices provide quantitative data on a patient’s renal function within minutes, which is useful in healthcare settings like emergency units and outpatient clinic settings, or even in the home [13]. Although recent studies have used POC devices to measure renal function, and reported promising results when comparing the accuracy of kidney function values, POC devices have yet to be tested outdoors [13–15]. Were the POC device provide accurate outdoor measurements, its usefulness for field studies of outdoor workers would increase.

Finally, despite the suspected strong role of occupational factors as determinants of CKDu in Hispanic worker populations, which typically are collected by means of questionnaires, there is a lack of survey questions available for researchers. However, the Second Central American Survey of Working Conditions and Health (II ECCTS by its Spanish acronym) conducted in 2018 among 9000 formal and informal workers in all six Spanish-speaking countries in Central America (Costa Rica, El Salvador, Guatemala, Honduras, Nicaragua, and Panama) developed a supplemental “Heat/Kidney Disease” module that included items specifically targeting the suspected CKDu risk factors. This module has not been tested among other groups of Hispanic workers that may be at risk for CKDu, such as Hispanic outdoors workers in the U.S.

In brief, the aim of this study was to examine the feasibility of implementing the DEGREE protocol, in combination with outdoor POC testing for serum creatinine and the application of a new module on risk factors for CKDu in Hispanic outdoor workers in order to determine their feasibility in the field and the usability for future research.

## Methods

A cross-sectional study was conducted in a convenience sample of 50 Houston-based Hispanic outdoor workers, recruited from the Health and Safety Council (HASC) facility in Pasadena, Texas. This study began in late fall 2017, after approval by the University of Texas Health Science Center at Houston Committee for the Protection of Human Subjects. HASC is a fully staffed medical and worker training facility serving contractors in the greater Houston area to where employers send their workers for various pre-employment and post-placement activities, such as: a) required and periodic medical evaluations, including laboratory testing; b) drug testing; c) respirator medical evaluations and fit testing; and d) mandated worker training sessions. A large proportion of the several hundred workers seen daily at HASC are Hispanic and work outdoors, primarily in construction. HASC allowed the authors to use their facility to approach potential participants, and for participant interviews and sample collection.

The team consisted of two Spanish-speaking research members, and a trained phlebotomist. A table, chairs, blood drawing area/portable exam table, and refreshment area were set up outdoors in the facility parking area. Ambient temperature and humidity were measured outdoors with a standard anemometer station (Model 8552/8554 Q-Trak Plus IAQ Monitor, TSI Incorporated, Shoreview, MN) and readings were recorded at hourly intervals.

Participants were approached in the HASC waiting areas, the study was explained to them and, if interested, they were screened for eligibility criteria: 1) 18 years old and older; 2) performing manual labor outdoors for a minimum of 20 h a week (e.g., construction, landscaping, etc.); 3) Hispanic; 4) no prior diagnosis of kidney injury or disease; 5) no disease associated with CKD (e.g., diabetes, hypertension, glomerulonephritis); and 6) not taking any medications to treat kidney injury, hypertension, or other cardiovascular disease. All study participants were men. This is in line with current evidence suggesting that the majority of the population affected by CKDu are young men, between the ages of 20 to 50, who work in outdoor fields [16]. Thus, the demographic characteristics of our sample  were intended to represent those of the target population. While there is some variation by industry and occupations, by far, the most common working hours per week are 40 h. We did not expect the population of workers in our study to be full-time employees. To avoid recruiting workers who may have worked just a few hours, we required workers to have been working outdoors in manual labor at least 20 h (i.e., half of the standard).

Informed consent and interviewer-administered questionnaires were completed indoors.

Eligible participants were administered two questionnaires (either in English (*n* = 44, 89%) or in Spanish (*n* = 6, 11%)) by a member of the research team: the DEGREE protocol data collection form and the II ECCTS Heat/Kidney module. After completing the questionnaire, participants were escorted outside to have height (in stocking feet) and weight recorded using a stadiometer and digital scale. Three resting blood pressures were obtained in the semi-sitting position using an electronic blood pressure monitor (Omron™ Digital Blood Pressure monitor with cuff, Bannockburn, Illinois; this device is programmed to take three consecutive blood pressure measurements) in accordance with the DEGREE protocol. Participants who consented to participate completed the questionnaire, the anthropometrics measurements, and the blood testing during the same first encounter. Those who refused to participate did not complete an eligibility form as they were only asked if they would be interested to screen their eligibility to the study.

A single blood sample was also obtained outdoors, in order to test the performance of the POC device in hot environments, with results compared to standard isotope dilution mass spectrometry (IDMS) analysis by a certified commercial laboratory (LabCorp™, Pasadena, TX). In order to avoid testing discrepancies between blood samples, and drawing blood from the participant from two different locations, all blood samples were obtained venipuncture. Approximately 10 cc, or two teaspoons, of blood was obtained by an experienced phlebotomist from a forearm vein, with the participant in the semi-sitting position. The specimen was then placed in one lavender, one red, and one green top tubes, each labelled with the participant’s study ID. The lavender and one red top tube were prepared for transport to a local commercial laboratory (LabCorp™), as per their manual of procedures, for measurement of hemoglobin and kidney function indicators (creatinine, blood urea nitrogen (BUN)), using IDMS, as per the DEGREE protocol. Both tubes were stored in an empty cooler and taken to the lab at the end of each recruitment day. Results were ready for review between one and two business days. Blood from the green top tube was used for the POC creatinine measurement with a single-use cartridges handheld analyzer (i-STAT® Chem 8+ Point of Care Handheld Analyzer, Model 04 J60–20, Abbott Laboratories, Abbott Park, Illinois), following the manufacturer’s procedure, and results were immediately recorded. The total estimated participant time for all components (questionnaires, anthropometrics, and lab draw) was about 25 min.

Most items in the DEGREE protocol questionnaire and the “Heat/Kidney Disease” module have the advantage of a response frequency scales, but others have only a dichotomous Yes/No response. Both the heat module and the DEGREE questionnaires have been published elsewhere [1, 17]. For the POC device readings, the gold standard was defined as the central laboratory-obtained values for creatinine, electrolytes and eGFR.

Scatter plots of paired lab and POC values were plotted and a line of equality drawn. The Pearson correlation coefficient (*r*) between the two methods was calculated in order to determine the linear correlation between the two methods, which quantifies the degree to which two variables are related, but does not assess or imply the agreement between the two methods. Therefore, Bland-Altman plots were generated to assess agreement with the percent differences and means between both methods [18]. These plots help show when there is an increase in variability of the differences as the magnitude of the measurement increases, and can highlight anomalies and reveal overestimates or underestimates of one method over the other [19]. For this analysis, the IDMS was used as the gold standard. A paired t-test was used to calculate negligible bias between the measurements for the POC and IDMS. The Stata statistical package (Stata Corp, College Station, TX) was used for the analyses [20].

## Results

Recruitment was divided between two time periods. The first 29 participants were recruited in November and December 2017, referred to as the “colder period” (outside temperature range from 59.7 to 86 °F, with an average relative humidity of 64.9%, and an average heat index of 76.6 °F), and a second group of 26 participants in April and May 2018, referred to as the “hotter period” (73.2 °F to 89.8 °F, with an average relative humidity of 69.3%, and an average heat index of 88.3 °F). When calculating for heat index using the recorded temperatures and relative humidity, the highest reported heat index was 106 °F.

We invited these 55 participants to complete the DEGREE protocol and CKDu module, and undergo core measurements. However, five participants did not complete the blood testing. The five participants who were excluded from the comparison analysis completed IDMS lab analyses. However, they were unable to be part of the POC testing as the POC i-STAT device had not been operated correctly by the phlebotomist. Once we corrected this error, the POC i-STAT successfully delivered the remaining 50 readings. The age of the final sample ranged from 19 to 60 years, with an average age of 29 years. The most common occupation was scaffold builder (*n* = 12). The majority of men had a high school education, while less than 6% had a college or professional degree (Table 1). Most individuals did not answer the question about income. Unfortunately, issues of non-response related to survey questions on income have long been reported [21]. There is no unique reason behind people not reporting their income. Possible reasons include lack of knowledge or recall, question misunderstanding, or, simply, sensitivity issues related to disclosing information about income. Administration of the DEGREE and core measurements, excluding blood draw, averaged 10 min. All questions were straightforward and easily understood by the participants; completion rate for both surveys was above 98%. Administration of the “Heat/Kidney Disease” module averaged 5 min, and all questions were answered without any difficulty, need for clarification or reluctance to answer.

**Table 1:** DEGREE Protocol social demographics by season

Demographics	Colder Period (***n*** = 24) N (%)	Hotter Period (***n*** = 26) N (%)
*Age (years)*		
19–29	14 (58.3%)	16 (61.5%)
30–39	6 (25.0%)	7 (26.9%)
40–49	3 (12.5%)	1 (3.9%)
50–59	0 (0%)	2 (7.7%)
60 +	1 (4.2%)	0 (0%)
*Occupation*		
Boilermaker	3 (12.5%)	1 (3.9%)
Carpentry	0 (0.0%)	0 (0.0%)
Certified mechanic	1 (4.2%)	1 (3.9%)
Construction	1 (4.2%)	0 (0.0%)
Electrician	1 (4.2%)	2 (7.7%)
Foundation repair	0 (0.0%)	1 (3.9%)
Industrial painter	0 (0.0%)	4 (15.4%)
Insulation	3 (12.5%)	2 (7.7%)
Ironworker	0 (0.0%)	2 (7.7%)
Pipefitter	2 (8.3%)	2 (7.7%)
Refineries	3 (12.5%)	1 (3.9%)
Scaffold builder	6 (25.0%)	6 (23.1%)
Skilled worker	0 (0.0%)	1 (3.9%)
Technician	1 (4.2%)	0 (0.0%)
Welder	0 (0.0%)	2 (7.7%)
Other	1 (4.2%)	1 (3.9%)
*Education*		
Less than high school	4 (16.7%)	1 (11.5%)
High School	9 (37.5%)	14 (53.9%)
Some College	9 (37.5%)	8 (30.8%)
College Degree / Professional Degree	2 (8.3%)	1 (3.9%)
*Household Income*		
< $30,000	0 (0.0%)	0 (0.0%)
$30,000 - $50,000	3 (12.5%)	0 (0.0%)
$50,000+	3 (12.5%)	0 (0.0%)
Did not answer	18 (75.0%)	26 (100.0%)
*Interview language*		
English	22 (91.7%)	22 (84.6%)
Spanish	2 (8.3%)	4 (15.4%)

Table 2 compares the anthropometric and physical measures dictated on the DEGREE protocol by season. Overall, average BMI was 31.26 (SD = 6.745) and average blood pressure was 132 mmHg for systolic and 85 mmHg for diastolic (SD = 11.70, 10.58). When measurements were separated to distinguish those from the colder period (*n* = 24) and the hotter period (*n* = 26), there were no differences in BMI. However, average blood pressure was lower for those participants recruited during the hotter period than the colder period (*p*-value = 0.016 diastolic; *p*-value = 0.0001 for systolic).

**Table 2:** DEGREE Protocol Core Physical Measurements by season

	Colder Period (***n*** = 24)				Hotter Period (***n*** = 26)			
Measurements	Mean	SD	Range	***p***-Value	Mean	SD	Range	***p***- Value
Temperature (°F)	74.5	8.3	(59.7–86)	0.0002	82.3	4.7	(73.2–89.8)	< 0.0001
Height (cm)	176.3	6.6	(164–187)	0.56	175.5	7.4	(155.5–191)	0.58
Weight (kg)	97.9	25.0	(61.7–152.4)	0.39	93.4	18.5	(62.1–140.2)	0.23
BMI	31.4	7.3	(20.0–50.3)	0.47	30.3	5.5	(20.7–45.2)	0.32
Systolic BP (mmHg)	134.0	12.0	(111.0–161.3)	0.087	129.6	8.7	(115.3–147.3)	0.016
Diastolic BP (mmHg)	88.7	9.6	(63.7–108.0)	0.0005	80.8	9.0	(65.7–104.0)	0.0001

*SD* Standard deviation; *BMI* Body Mass Index; *BP* Blood pressure

Regarding the comparison between POC and IDMS measurements, we found that measurements of creatinine and blood urea nitrogen (BUN) correlated well across the range of temperatures, and had a slightly higher correlation coefficient during the hotter period (Table 3). Hemoglobin measurements also correlated strongly during the coldest outdoor temperatures and the hottest temperatures. Strong correlations were observed for hemoglobin, serum creatinine, and BUN (r = > 0.850).

**Table 3:** Comparison of IDMS measurements and Point-of-Care i-STAT CHEM8+ measurements by season

Blood **C**hemistries	Colder Period (***n*** = 24)					Hotter Period (***n*** = 26)				
	IDMS	iSTAT				IDMS	iSTAT			
	Mean (SD)	Mean (SD)	Percent Difference (%)	***r***	**Bias** Mean (95%CI)	Mean (SD)	Mean (SD)	Percent Difference (%)	***r***	**Bias** Mean (95%CI)
Hemoglobin (g/dL)	16.03 (1.21)	16.86 (1.12)	5.05	0.878	0.833 (0.60/1.06)	15.47 (1.220)	15.85 (1.221)	2.43	0.868	0.388 (0.14/0.64)
Creatinine (mg/dL)	0.88 (0.13)	0.89 (0.15)	1.13	0.896	0.013 (−0.018/0.045)	0.95 (0.1361)	0.965 (0.1788)	1.57	0.914	0.013 (−0.015/0.39)
BUN (mg/dL)	12.83 (3.82)	13.25 (4.34)	3.22	0.964	0.35 (− 0.049/0.74)	13.46 (3.08)	13.81 (3.86)	2.57	0.986	0.42 (−0.095/0.93)
eGFR (ml/min/1.73m^2^)	114.00 (15.18)	–		–		105.81 (18.21)	–		–	

*IDMS* Isotope dilution mass spectrometry; *SD* Standard deviation; *BUN* Blood urea nitrogen; *eGFR* Estimated glomerular filtration rate; *r = Pearson’s* correlation coefficient

**Fig. 1:**
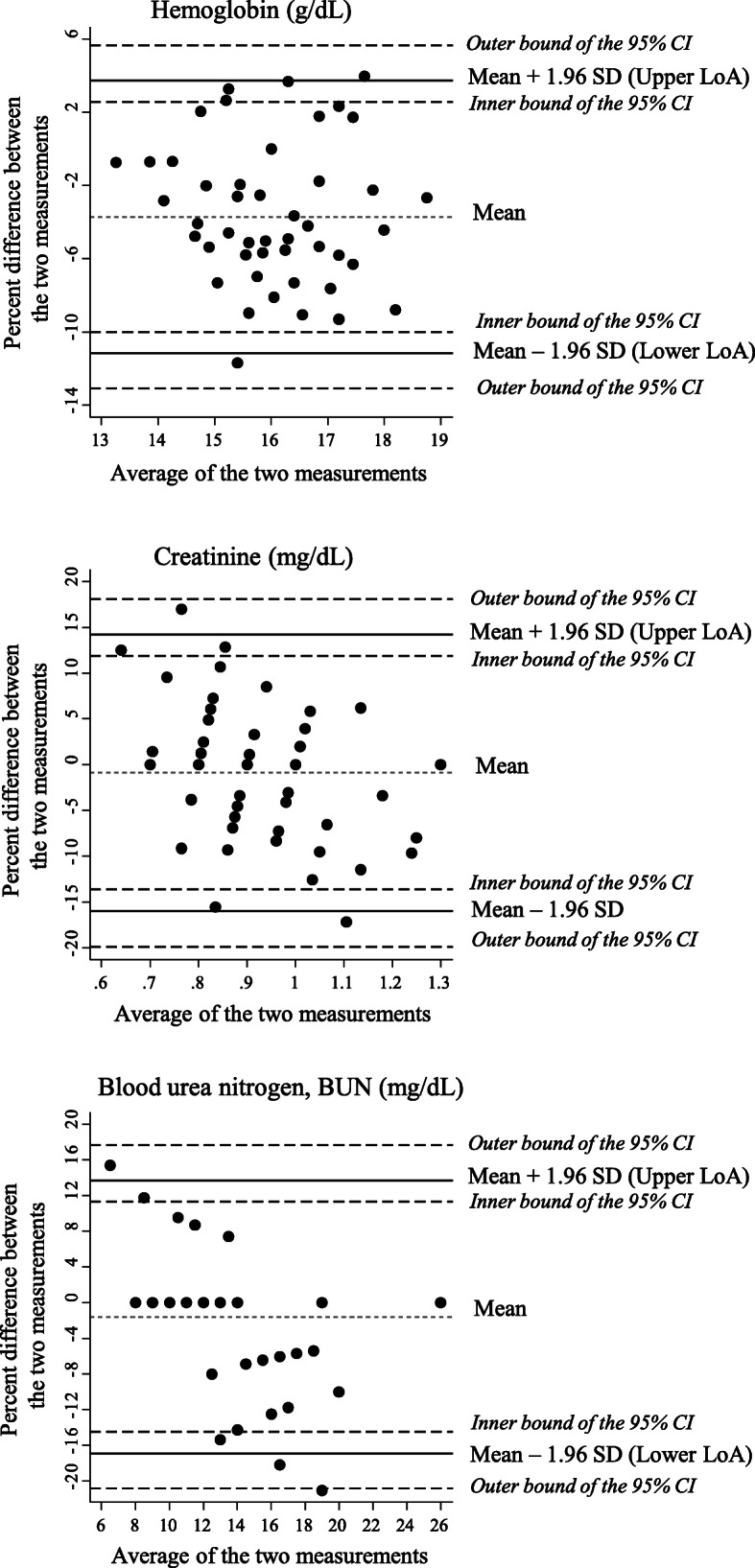
Bland-Altman plot of agreement between the measurements from IDMS and POC i-STAT (n=50). *LoA: Limits of Agreement. CI: Confidence Interval*

The Bland-Altman plot for hemoglobin demonstrated a bias of − 3.58% and an agreement range of − 11.43 to 4.27%, suggesting that, on average, the POC provides measurements 3.58% higher than those obtained from the IDMS analysis. Serum creatinine indicates a near negligible bias of − 0.18%. Serum BUN has a bias of − 0.72% that is constant for lower averages of BUN (compared to a − 1.59% bias with all 50 samples). The hemoglobin plot was the only plot to show no evident trend (Fig. 1). Additionally, given our relatively small sample, we calculated 95% confidence intervals for the Bland-Altman Limits of Agreement (LoA) [22]. Since Bland-Altman LoAs are usually considered as a pair of bounds, we treated them as such when calculating their confidence limits. The confidence intervals indicate where the LoAs in the population would lie. The farther from the mean, the farther the LoA bound in the population may be from the observed LoA bound. In our figures, the 95% CIs indicate that the LoAs in the population would be near the mean of differences.

The POC device generally worked well outdoors, with one exception. POC tests performed immediately after the device dysfunction required to be brought indoors to allow the device to warm up or cool down as needed. This occurred on the 2 days where the temperature was the lowest (below 65 °F) or highest (above 85 °F), and affected a total of 4 tests.

## Discussion

One major strength from our study is that all blood samples were collected from the same site, i.e., the forearm. Thus, all three blood vials collected from each participant contained the same whole blood source for simultaneous POC and IDMS testing. Executing this also avoided drawing blood from the participant two different times from two different places (forearm and finger). One recent study did report POC testing overestimated creatinine by 22% compared to laboratory testing using a distinct method of blood sample collection (fingerprick for POC and venipuncture for IDMS) under tropical field conditions [23]. Thus, an adjustment factor of 0.7775 was applied to all POC creatinine values for a later study analyzing cross-shift percent change in eGFR from 105 sugarcane workers in Guatemala [24]. There may be an advantage to using a venipuncture blood sample rather than a fingerstick sample due to blood volume and testing efficiency. Recently, a study performed analytical and clinical comparability of POC devices with a central laboratory reference for creatinine and found that the i-STAT device showed the highest clinical concordance with the reference standard (Kappa: 0.94) and had the smallest average analytical error (6%) for creatinine when using a whole blood sample [25].

There were some limitations. Since all blood samples completed by venipuncture, we did not examine accuracy of venipuncture versus fingerprick results when using a POC device. The POC allows for both fingerstick and venipuncture blood testing. Studies that have used POC testing rely on a fingerprick for the POC device and venipuncture for laboratory testing. A study done in Nicaragua found POC testing for creatinine demonstrated acceptable repeatability, excellent sensitivity (100%) and modest specificity (79%) using blood samples from two different sources (forearm for Jaffe kinetic method with a Roche Cobas Integra 400 analyzer and fingerstick for POC) [26]. However, there are no available studies that compare the accuracy of solely using fingerprick capillary blood for comparable POC and IDMS results. Nevertheless, if future studies can find the accuracy of using fingerprick blood, it may be a preferable method over venipuncture due to lower cost of equipment, faster laboratory testing, and a less invasive blood draw.

Only a few of the outdoor temperature ranges were as warm or hot as those expected in the Central American agricultural fields. Central America has a tropical humid climate with no real winter; even the coldest month averages above 64.4 °F, with summers of 80.6 °F to 89.6 °F, with a relative humidity between 75 and 85%. When using the heat index to measure the heat index temperature, the temperature in the agricultural areas can increase significantly. A temperature in Central America of 90 °F with a relative humidity of 80% can result in a heat index temperature of 113 °F [27]. The highest reported heat index for this study was 106 °F. Thus, it may be useful to retest the POC at heat indices that are closer to 113 °F. This is important given that cartridge testing with the POC we used was only possible within a range of outdoor temperatures between 65 and 85 °F. However, only a few minutes were needed to cool the device and reinstate the testing function, with no reading bias observed after this was done. Certainly, more studies are needed to determine what obstacles can be encountered with POC devices in outside settings (e.g., to test if readings are stable and accurate despite long term exposure to heat) and what remedies can be developed to overcome those issues. For instance, repeating our study by supplementing the POC sensor with a small cooler in order to cool down the POC sensor, would help determine the effectiveness of the POC device in the field and in hotter temperatures.

## Conclusions

Implementation of the DEGREE protocol and CKDu module was straightforward and well understood. Participants had no difficulty understanding the questions or procedures that needed to be completed (i.e., blood pressure, blood draw), in either English or Spanish. We did not encounter any major problems with POC testing for renal function or hemoglobin measurements. Results for BUN, creatinine and hemoglobin correlated well with those obtained from IDMS.

It may be beneficial to conduct more research studies with a larger sample population to better determine the feasibility of using the DEGREE protocol, POC testing, and “Heat/Kidney Disease” module. This was a pilot study. As such, sample size and power calculations were not the major consideration; instead, our study focused on the feasibility of measuring the accuracy of POC creatinine testing against the gold standard of lab-based testing. In this regard, our sample of 50 persons should be sufficient to answer the study aims. For example, a recent pilot study had a study population of 60 participants to study genetic biomarkers in blood to distinguish and identify CKDu from CKD as well as healthy populations from CKDu endemic and non-endemic areas of Sri Lanka [28]. In addition, as part of the eligibility survey, recruited participants responded to not having a diagnosis of CKD or being aware of any cardiovascular disease (e.g., hypertension). None of the participants in our study had a previously known elevated creatinine level. Next steps in future studies should include measuring the POC test accuracy in a larger sample with individuals who do have known elevated creatinine levels or suffer from kidney disease. Given the extremely high mortality and morbidity reported with this disease, efforts to identify baseline of normal and reduced eGFRs, faster in-field serum measurement testing that circumvents the need for specimen preservation and transport to an offsite laboratory, and common risk factors associated with CKDu can help researchers prevent future cases and provide care for those affected. Future studies should also focus on testing the comparability of POC and laboratory testing using only one type of blood source.

## Data Availability

The datasets generated during this study are available from the corresponding author on reasonable request.

## Abbreviations

II ECCTS: Second Central American Survey of Working Conditions and Health
CKD: Chronic kidney disease
CKDu: Chronic kidney disease of unknown origin
eGFR: Estimated glomerular filtration rate
DEGREE: Disadvantaged Populations Estimated Glomerular Filtration Rate (eGFR) Epidemiology Study
IDMS: Isotope dilution mass spectrometry
POC: Point of care
